# Genomic Tools and Selective Breeding in Molluscs

**DOI:** 10.3389/fgene.2018.00253

**Published:** 2018-07-18

**Authors:** Christopher M. Hollenbeck, Ian A. Johnston

**Affiliations:** ^1^School of Biology, Scottish Oceans Institute, University of St Andrews, St Andrews, United Kingdom; ^2^Xelect Ltd, St Andrews, United Kingdom

**Keywords:** aquaculture, genomics, SNP genotyping, heritability, marker assisted selection, genomic selection, molluscs, selective breeding

## Abstract

The production of most farmed molluscs, including mussels, oysters, scallops, abalone, and clams, is heavily dependent on natural seed from the plankton. Closing the lifecycle of species in hatcheries can secure independence from wild stocks and enables long-term genetic improvement of broodstock through selective breeding. Genomic techniques have the potential to revolutionize hatchery-based selective breeding by improving our understanding of the characteristics of mollusc genetics that can pose a challenge for intensive aquaculture and by providing a new suite of tools for genetic improvement. Here we review characteristics of the life history and genetics of molluscs including high fecundity, self-fertilization, high genetic diversity, genetic load, high incidence of deleterious mutations and segregation distortion, and critically assess their impact on the design and effectiveness of selective breeding strategies. A survey of the results of current breeding programs in the literature show that selective breeding with inbreeding control is likely the best strategy for genetic improvement of most molluscs, and on average growth rate can be improved by 10% per generation and disease resistance by 15% per generation across the major farmed species by implementing individual or family-based selection. Rapid advances in sequencing technology have resulted in a wealth of genomic resources for key species with the potential to greatly improve hatchery-based selective breeding of molluscs. In this review, we catalog the range of genomic resources currently available for molluscs of aquaculture interest and discuss the bottlenecks, including lack of high-quality reference genomes and the relatively high cost of genotyping, as well as opportunities for applying genomics-based selection.

## Introduction

**Figure 1:**
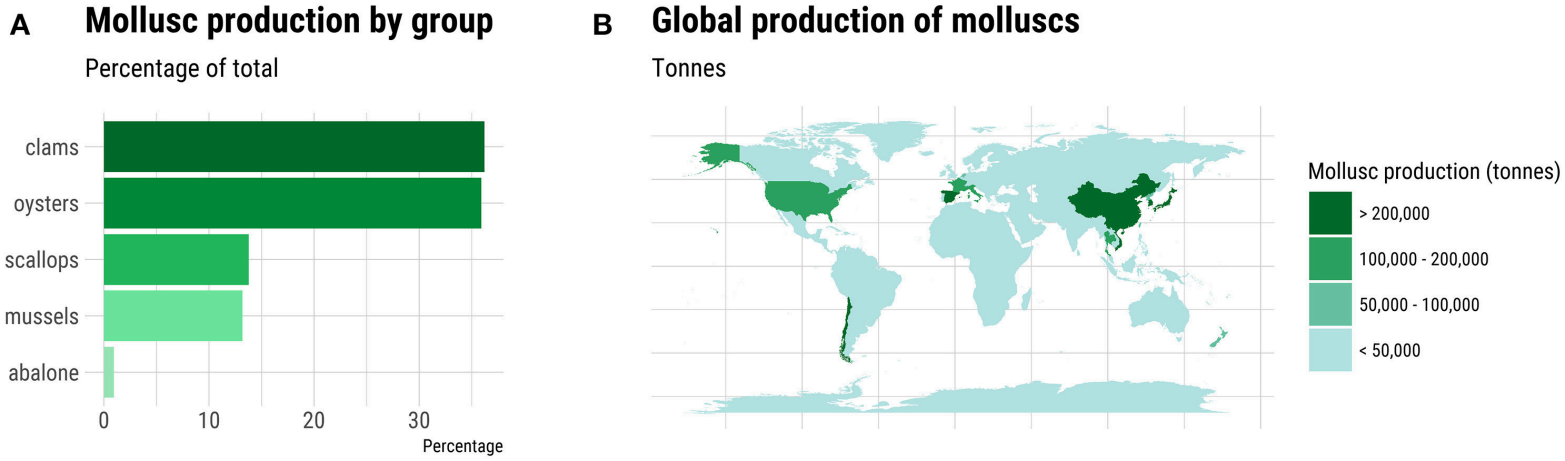
Global production of molluscs in 2015. **(A)** The percentage of total production for each major group of farmed molluscs. **(B)** Geographic distribution of major mollusc producing countries. Source: FAO (2017).

Molluscs comprise a diverse Phylum comprising ten classes and some 85,000-extant species. The majority of the 80-individual species farmed come from the Classes Bivalvia, Cephlapoda, and Gastropoda. Bivalves, namely clam, oyster, scallop and mussel species, dominate in terms of tonnage and economic value (Figure 1A). In total, more than 16 million metric tons of molluscs were farmed worldwide in 2015 with Asia dominating production (Figure 1B; FAO, 2017). The life cycle has been closed for relatively few species, but this does include some of the most valuable species (Table 1). The majority of production, even for the species listed in Table 1, occurs in extensive aquaculture and is dependent on spat harvested from the wild (Boudry, 2009; Astorga, 2014). However, despite its cost-effectiveness, farming based on the collection of wild seed is subject to environmental risk, including depletion of local stocks, disease outbreaks (Boudry et al., 1997, 2004) and the adverse effects of climate change (Waldbusser et al., 2015). The future for industrialized aquaculture of molluscs undoubtedly lies with closing the life cycle of more species and in the development of hatcheries that can provide sufficient production of seed for on-growing in the sea, while allowing selective breeding to improve commercially important traits such as growth, meat yield, disease resistance, and stress tolerance (Boudry et al., 1997; Camara and Symonds, 2014).

**Table 1:** Top ten cultured mollusc species by value in 2015.

**Species**	**Common name**	**Value (million USD)**	**Production (tons)**
*Crassostrea gigas*	Pacific oyster	3880.89	5178707
*Ruditapes philippinarum*	Manila clam	3708.93	4049540
*Mytilus chilensis*	Chilean mussel	1711.4	208707
*Sinonovacula constricta*	Constricted tagelus	714.34	793708
*Anadara granosa*	Blood cockle	576.82	441303
*Perna canaliculus*	Greenlip mussel	494.86	76811
*Patinopecten yessoensis*	Yesso scallop	475.15	251907
*Mytilus edulis*	Blue mussel	325.05	192271
*Argopecten purpuratus*	Peruvian calico scallop	217.97	25988
*Meretrix lusoria*	Japanese hard clam	141.03	64060

Source: FAO (2017)

**Figure 2:**
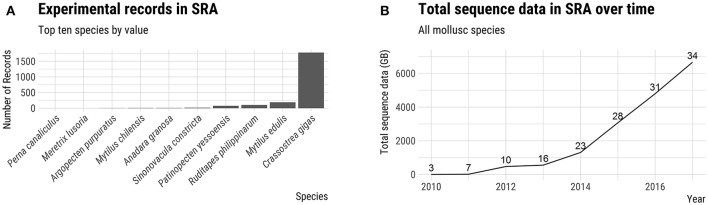
Genomic resources of farmed molluscs. **(A)** Number of experimental records in NCBI's Short Read Archive (SRA) for top 10 farmed mollusc species by value. **(B)** Total number of bases (GB) in the SRA for all species of farmed molluscs. The number above the line for each year indicates the number of farmed mollusc species represented in the SRA database. Source: FAO (2017) and NCBI SRA.

Advances in sequencing technology have provided a powerful set of genetic tools which can facilitate the establishment of hatchery-based breeding programs (Yáñez et al., 2015). In recent years, six molluscs of aquaculture significance have had their genomes sequenced and annotated (see Discussion below). Although this compares unfavorably with the more than 20 genomes of aquaculture finfish sequenced, the amount of publically available DNA sequence data for molluscs, as measured by the total number of bases in NCBI's Short Read Archive (SRA) database, has increased dramatically in the last 2 years (Figure 2). The generation of genomic resources for non-model species has been particularly accelerated by the advent of cost effective reduced-representation genome sequencing techniques such as genotyping-by-sequencing (GBS; Elshire et al., 2011) and RAD sequencing (RAD-seq; Baird et al., 2008). The aim of this review is to provide an overview of the genetics and life history of the major farmed molluscs and to discuss the implications for hatchery-based selective breeding. We present a survey of the current state of selective breeding with an emphasis on relevant genetic parameters. Finally, we discuss the application of genomic resources and the use of genetic markers in breeding programs, highlighting some of the bottlenecks and opportunities for increasing production using these new tools.

## Implications of life history characteristics for selective breeding

Several life history characteristics of molluscs have major implications for selective breeding, including high fecundity and the incidence of hermaphroditism with the potential for self-fertilization. Pacific oysters, for example, can produce tens to hundreds of millions of eggs in a single spawning, resulting in potentially very large family sizes (Quayle, 1988). Diverse reproductive strategies have been documented (Collin, 2013). While most species of clams, mussels, and abalone have separate sexes, clams of the genus Venerupis, Mercenaria, Mya and Spisula, oysters of the genus Crassostrea and Ostrea and mussels including Mytilus can be to various degrees sequential hermaphrodites, whereas most farmed species of scallops are simultaneous (functional) hermaphrodites. Many species display a combination of strategies, with Pacific oysters typically maturing first as males then changing to females (protandric hermaphroditism), but they can also be functionally hermaphroditic at low frequency, and sex-reversal (female to male) can occur depending on environment and food supply (Guo et al., 1998). Individual European flat oysters can go through two or three sex reversal cycles in each spawning season (Joyce et al., 2013). Functional hermaphroditism produces a proportion of self-fertilized gametes, which can quickly elevate levels of inbreeding. For some species of scallops, rates of self-fertilization can average ~20 percent within particular families, and in addition to the effect on levels of inbreeding, failing to account for self-fertilization can result in biased estimates of genetic parameters such as heritability (Martinez, 2007). Finally, extremely high fecundity appears to be a critical factor in explaining several genetic characteristics of mollusc species that directly influence selective breeding (see below).

## Implications of genetic characteristics for selective breeding

Decades of genetic studies involving molluscs have revealed a number of characteristics that differentiate molluscs from other aquaculture species. In particular, molluscs have been shown to exhibit extremely high levels of genetic diversity (Peñaloza et al., 2006; Zhang et al., 2012; Romiguier et al., 2014; Wang et al., 2017), deviations from Hardy-Weinberg proportions in wild populations (i.e., Zouros and Foltz, 1984 and references therein), non-Mendelian segregation of marker loci in pair crosses (Launey and Hedgecock, 2001), high incidence of null alleles at genetic markers (Hedgecock et al., 2004), and correlations between fitness traits and levels of heterozygosity (David, 1998).

## High genetic diversity

Molluscs exhibit extremely high levels of nuclear genetic diversity relative to many other taxa based on a variety of metrics (English et al., 2000; Bazin et al., 2006; Peñaloza et al., 2006; Sauvage et al., 2007; Zhang et al., 2012; Harrang et al., 2013; Romiguier et al., 2014; Wang et al., 2017), although the ultimate cause of such diversity is not well-understood (Plough, 2016). The neutral theory of evolution predicts that the steady-state level of heterozygosity should scale positively with effective population size (*N*_*e*_) and mutation rate (Nei and Li, 1979), and it is likely that both factors contribute to the extreme levels of variation observed. The extreme fecundity of molluscs and a tendency toward broadcast spawning (which promotes homogeneity of populations) would favor large effective population sizes, although the high variance in reproductive success commonly observed in molluscs may considerably reduce *N*_*e*_ relative to census size (Hedgecock, 1994; Hedgecock and Pudovkin, 2011). In addition, mutation rates for molluscs may be elevated due to the number of meiosis events necessary to produce millions of eggs during reproduction. For example, Plough et al. (2016) estimated that the mutation rate in the Pacific oyster was 90-fold higher than in Drosophila based on the number and effects of lethal alleles segregating in wild pair crosses. Observations of numerous and highly active transposable elements in molluscan genomes, as well as a high incidence of structural variation, also support the hypothesis of an elevated mutation rate (Zhang et al., 2012; Plough, 2016).

## Segregation distortion and genetic load

Since the earliest studies utilizing electrophoretic genetic markers on wild populations of molluscs, two of the most commonly observed phenomena have been deficiencies of heterozygous genotypes relative to expectations of Hardy-Weinberg equilibrium (HWE) and deviation from expected Mendelian ratios (segregation distortion) in pair crosses. These phenomena have been observed across the major groups of cultured molluscs and with a variety of marker types, including allozymes (Zouros and Foltz, 1984; Mallet et al., 1985; Gaffney et al., 1990; Beaumont, 1991; Toro and Vergara, 1995), microsatellites (Bierne et al., 1998; Launey and Hedgecock, 2001), and SNPs (Peñaloza et al., 2006; Vervalle et al., 2013; Hedgecock et al., 2015; Niu et al., 2017). The earliest explanations for these phenomena implicated null alleles, aneuploidy, inbreeding, cryptic population structure (Wahlund effect), or some form of selection (Beaumont, 1991). Null alleles, caused by mutations that produce truncated or inactive allozymes (discussed in Gaffney et al., 1990) or reduce primer binding affinity for amplification of PCR-based markers (McGoldrick et al., 2000), are a likely explanation for at least part of the observed heterozygote deficiencies, and it has been shown in some cases that redesign of PCR primers has eliminated the significant heterozygote deficiencies initially observed (e.g., Hare et al., 1996). Conversely, the incidence of null alleles is often insufficient to account for the observed level of heterozygote deficiency (Beaumont, 1991), and studies that have accounted for null alleles by tracking them through pedigrees have still observed considerable segregation distortion (McGoldrick et al., 2000; Launey and Hedgecock, 2001). A similar argument applies to aneuploidy, which has not typically been observed at high enough levels in molluscs to account for the degree of deviation from HWE or Mendelian ratios (Thiriot-Quiévreux et al., 1992). Further, the fact that segregation distortion is commonly observed in controlled pair crosses (Beaumont, 1991) has eliminated population-level inbreeding and Wahlund effects as a single explanation for the phenomena. Based on these observations, some form of selection is the most likely cause (Beaumont, 1991; Toro and Vergara, 1995). Inbred crosses of European oyster (Bierne et al., 1998) and Pacific oyster (Launey and Hedgecock, 2001) have shown large homozygote deficiencies at microsatellite loci consistent with selection against a large genetic load of deleterious recessive mutations: 15–38 per genome for *O. edulis* and 8–14 per genome for *C. gigas*. Both studies also found that genotypic proportions were typically Mendelian at early larval stages and became increasingly distorted with age, which strongly supported viability selection as a cause of the distortions over pre-zygotic possibilities such as meiotic drive. QTL mapping of segregating lethal alleles in inbred *C. gigas* has demonstrated large homozygote deficiencies and evidence of at least 14–15 recessive or partially recessive viability QTL per genome, and estimated that mortality attributed to viability selection up to the spat stage was ~96%, which was consistent with observations of mortality in hatchery populations (Plough and Hedgecock, 2011). Most recently, Plough et al. (2016) found mortality attributable to viability selection (~99%) in wild crosses of *C. gigas* to be as high as that observed for inbred crosses, except in this case the genetic load was expressed primarily as selection against additive or partially dominant mutations, resulting in a deficit of heterozygotes. These experimental results from oysters, along with evidence of extremely high levels of genetic diversity in other molluscs, provide a compelling case that a high genetic load is largely responsible for heterozygote deficits in wild populations and segregation distortion in pair crosses (Plough, 2016). One difficult to resolve aspect of this discussion is the fact that there is considerable heterogeneity in the literature with respect to these phenomena, whereby at times segregation distortions in pair crosses are extreme (Launey and Hedgecock, 2001; Wang et al., 2004; Plough and Hedgecock, 2011; Plough et al., 2016) and at other times small or non-significant (Wang et al., 2005; Gutierrez et al., 2017) for the same species. Similarly, reports of segregation distortion sometimes primarily involve homozygote deficits (Launey and Hedgecock, 2001; Plough and Hedgecock, 2011) and at other times primarily heterozygote deficits (Peñaloza et al., 2006; Plough et al., 2016). An attractive feature of the genetic load hypothesis is that it can account for this heterogeneity by way of the fact that expression of genetic load is highly context dependent (Barrett and Charlesworth, 1991; Pekkala et al., 2012). Differences in the overall levels of diversity (and the amount of genetic load) between populations (Lohr and Haag, 2015), the level of inbreeding in a population or individuals in a cross (Barrett and Charlesworth, 1991), levels of dominance and epistasis of the genes involved (Whitlock and Bourguet, 2000), developmental timing of expressed lethals (Plough and Hedgecock, 2011), and the extent to which lethal mutations have been purged from a population (Crnokrak and Barrett, 2002) will all influence the way that genetic load is expressed. For instance, if genetic load in Pacific oyster consists of both partially dominant and recessive mutations, this explains why pair crosses from wild individuals (Plough et al., 2016) would exhibit heterozygote deficiencies (due to initial expression of partially dominant lethals) and inbred crosses (Launey and Hedgecock, 2001; Plough and Hedgecock, 2011) would largely exhibit homozygote deficiencies due to recessive lethals, as partially dominant lethals would likely be purged in previous generations of inbreeding.

## Molluscan genetics and selective breeding

A high genetic load, combined with high fecundity, provides the opportunity for large variance in reproductive success among breeders (Hedgecock and Pudovkin, 2011), a phenomenon that has often been observed in molluscs in hatchery settings (Boudry et al., 2002; Lallias et al., 2010). The uneven contribution of parents has the potential to rapidly decrease the *N*_*e*_ of the breeding population, which can lead to both loss of genetic diversity through genetic drift and rapidly increase the level of inbreeding. Both can reduce the long-term response to selection (Lacy, 1987; Meuwissen and Woolliams, 1994). Inbreeding is of particular concern for bivalve aquaculture because inbreeding load—the rate at which fitness declines with increases in inbreeding coefficient (Charlesworth and Willis, 2009)—is likely higher for bivalves than for other animals (Plough, 2016). In support of this, inbreeding depression, typically characterized by reduced growth and increased mortality at early life stages, has been well-documented in cultured molluscs, including in European flat oyster (Bierne et al., 1998; Naciri-Graven et al., 2000), Pacific oyster (McGoldrick and Hedgecock, 1997; Evans et al., 2004), Pacific abalone (*Haliotis discus*; Deng et al., 2005), and catarina scallop (*Argopecten ventricosus*; Ibarra et al., 1995). Careful control of inbreeding is thus essential for molluscan selective breeding, and thus methods for optimizing the rate of genetic gain while constraining the rate of inbreeding such as optimal contribution selection (Meuwissen, 1997) and mate selection (Kinghorn, 2011) are important to consider. A further discussion of inbreeding management in aquaculture can be found in Nguyen (2016).

The second important consideration for genetic improvement is whether evidence for non-additive genetic variance for survival and other traits warrants a strong consideration of crossbreeding inbred lines to exploit heterosis as a more optimal breeding strategy than selection without inbreeding, which has become the standard means of genetic improvement for most finfishes (Gjedrem and Rye, 2016). Here, there are both genetic and practical considerations. From a genetic perspective, the more non-additive genetic variation that exists for a trait, the more benefit can be gained by exploiting heterosis. However, it is also important to consider the nature of the non-additive variance. In particular, quantitative genetic theory demonstrates that it is only when there is some level of overdominance with respect to the trait that crossing inbred lines can achieve what selection without inbreeding cannot (Falconer and Mackay, 1996). In general, while direct evidence for heterosis has been observed in molluscs (Cruz and Ibarra, 1997; Hedgecock and Davis, 2007; Deng et al., 2010), other studies have found little or no evidence for heterosis with multiple strains and production systems (e.g., In et al., 2017), and overall there is little evidence for overdominance of relevant traits. Both in studies of heterozygote fitness correlations and direct investigations of heterosis, results have been more consistent with a dominance or epistatic model of heterosis (Hedgecock et al., 1995; David, 1998; Hedgecock and Davis, 2007), suggesting that inbreeding and crossing could be a sub-optimal strategy. One important practical consideration is the difficulty in producing and maintaining inbred lines of molluscs for crossbreeding. Molluscs are not as easily grown, maintained, and propagated as crop species such as maize that have benefited from crossbreeding (Hallauer, 2009), nor is it often possible to crossbreed already established inbred lines or breeds, as is often beneficial in cattle (Gregory and Cundiff, 1980). Thus, while some success has been made with inter-specific hybridization in species such as abalone (Lafarga de la Cruz and Gallardo-Escárate, 2011), the practical difficulties in maintaining and testing enough inbred lines for effective genetic improvement is a barrier for crossbreeding in most molluscs. In summary, selective breeding with effective inbreeding control remains the primary strategy for genetic improvement in molluscs. As is discussed in the following sections, current research indicates large amounts of additive genetic variance for production traits across all important groups of molluscs, and selective breeding programs to date have experienced promising levels of genetic gain per generation. Hereafter, we will discuss selective breeding as selection with control of inbreeding.

## Current status of selective breeding programs

### Traits and genetic parameters

The aim of breeding programs is to improve one or more traits of commercial importance, such as growth rate, yield, shell pigmentation, temperature tolerance, and disease resistance. Because profitability is one of the key measures of success for breeding programs, existing programs for molluscs have often established breeding goals based on input from industry (Kube et al., 2011; Camara and Symonds, 2014), and the breeding goal often consists of multiple economically important traits to improve simultaneously. Once the traits for improvement have been identified, developing an understanding of the genetic basis of the traits is a crucial step toward implementing selective breeding. Three particularly important genetic parameters of interest are heritability, genetic correlations between traits, and interactions between genotypes and environment (Falconer and Mackay, 1996; Boudry, 2009).

#### Heritability

Heritability is a measure of the extent to which a phenotype is genetically determined. Narrow-sense heritability, in particular, determines the expected response of a trait to selection. In general, it is difficult to make generalizations about heritability of particular traits because estimates of heritability for the same trait can differ greatly depending on genetic diversity, life stage, and environmental conditions (Gjedrem and Thodesen, 2005). For example, Zheng et al. (2004) found realized heritabilities for larval growth rate in two hatchery stocks of bay scallops (*Argopecten irradians*) spawned and reared in common conditions to be 0.015 and 0.511 (more than a 30-fold difference), presumably due to very different levels of genetic diversity in the two stocks. Drawing general conclusions from heritability estimates is also confounded by the fact that heritability for the same trait can change dramatically depending on the life stage at which the trait is measured. For example, heritability for shell length was shown to increase with age in red abalone (*Haliotis rufescens*; Jonasson et al., 1999), but decreased in bay scallops (Zheng et al., 2004), and there is little to no genetic correlation between larval and juvenile traits in Pacific oyster (Ernande et al., 2003). Overall, there are two noteworthy trends. First, most traits of commercial interest in molluscs have been found to have moderate to high narrow-sense heritability (Supplemental File 1), suggesting that additive genetic variance for production traits is generally sufficient for selective breeding. Second, genetic markers are increasingly being used to improve estimates of heritability using family variance components, because molecular parentage assignment allows for families to be reared in common environment, eliminating a potentially confounding source of environmental variation (Vandeputte et al., 2004; Nguyen et al., 2014; Kong et al., 2015).

#### Genetic correlations

Knowledge of genetic correlations can often be used to advantage in breeding programs, when a trait that is difficult to measure can be improved by selecting for a correlated trait that is easier to measure (Falconer and Mackay, 1996). Examples in molluscs include genetic correlations between growth and difficult-to-measure traits such as meat yield (de Melo et al., 2016), shell strength (Camara and Symonds, 2014), and disease resistance (Dégremont et al., 2015). A more extensive survey of genetic correlations in molluscs can be found in Supplemental File 1.

#### Genotype-by-environment interactions

Often individuals from centralized breeding programs will be distributed and grown out in many different environmental conditions, and individuals with a particular genotype may exhibit superior phenotypic performance in one environment but not another (Falconer and Mackay, 1996). If significant GxE interactions are present, it may be beneficial to develop multiple selected lines with superior performance in particular environments (Sheridan, 1997). Unfortunately, studies measuring GxE interactions are still mostly lacking for most cultured molluscs (see Supplemental File 1). Experiments in Pacific oyster have revealed weak or no GxE interactions for growth (Evans and Langdon, 2006) and survival (Dégremont et al., 2007), albeit in relatively restricted geographic regions. In contrast, a study involving Eastern oyster found large, significant GxE interactions for both yield and cumulative mortality (Proestou et al., 2016). Breeding studies of silver-lipped pearl oyster have shown minimal GxE interactions for growth (Kvingedal et al., 2010), but larger effects for pearl quality traits (Jerry et al., 2012).

### Response to selection

**Figure 3:**
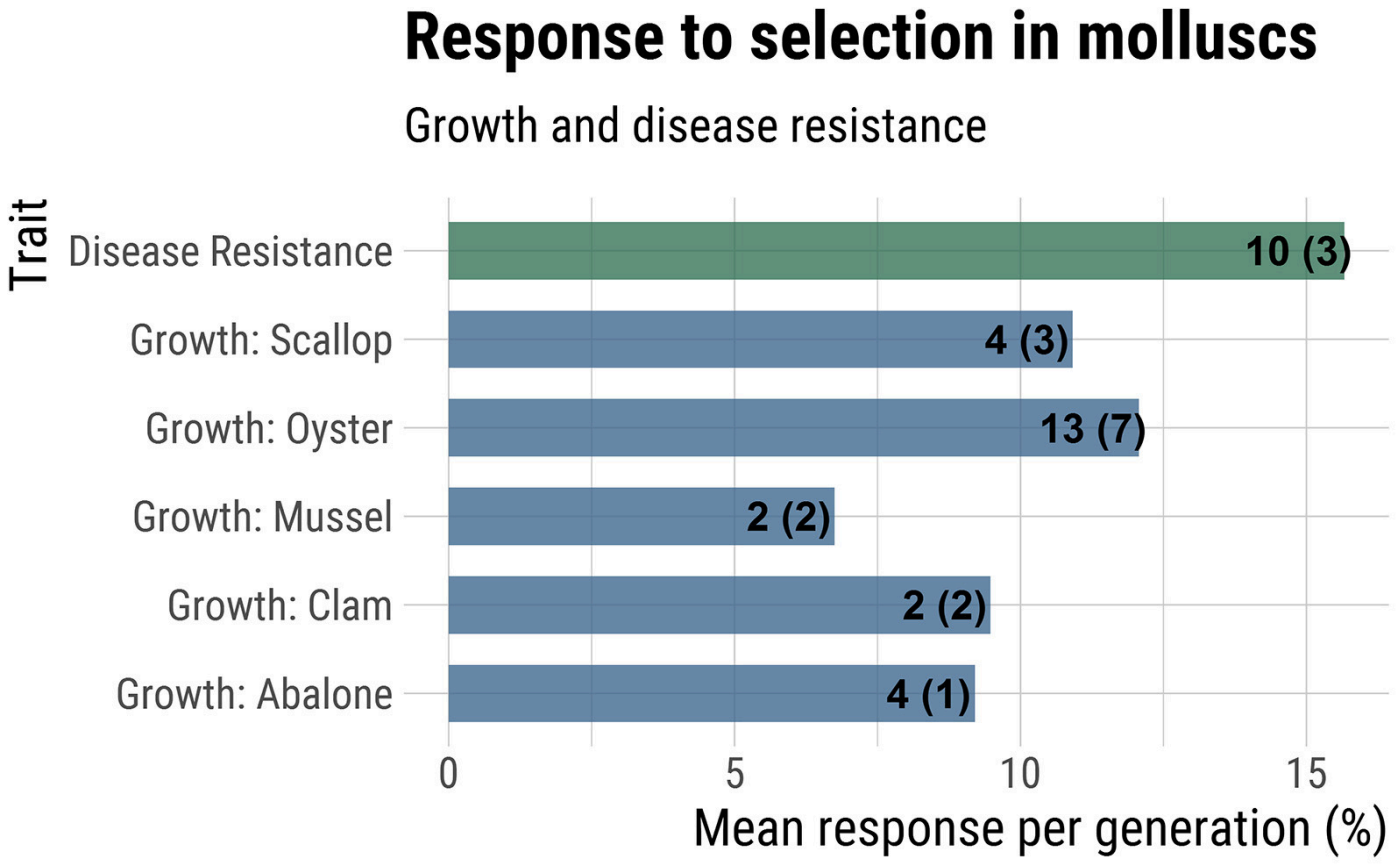
Response to selection for growth and disease resistance. Mean response to selection (% increase in additive genetic value over base population mean) is reported on the x-axis. The numbers at the end of each bar indicate the total number of generations of selection used in the calculation and the number of studies considered (in parentheses). A more complete summary of the data collected can be found in Supplemental File 1, under the tab “Response to Selection”.

Response to selection for production-related traits may be higher for molluscs than finfish due to their higher fecundity, which allows for a greater intensity of selection (Gjedrem and Thodesen, 2005; Gjedrem and Robinson, 2014). For molluscs, there are numerous studies measuring realized response to selection, which involves phenotypic comparison of selected lines against unselected controls. The majority of studies report response after a single-generation of mass selection, although a few studies encompass multi-generational mass selection (Dégremont et al., 2015) or family-based selection (Liu et al., 2015; de Melo et al., 2016). An extensive but non-exhaustive survey of the literature reporting response to selection for production traits in molluscs revealed that the average response to selection per generation for growth ranged from 6.8% for mussels to 12.1% in oysters (Figure 3). Averaged across studies for all mollusc taxa, the average response to selection for growth was 10.6% per generation. Average response to selection for disease resistance traits was even higher at 15.7% (Figure 3).

### Selective breeding programs

There are several strategies for selective breeding, and the optimal choice depends on a variety of factors, notably the genetic properties of the traits to be selected and the associated costs of implementing a particular strategy. Typically, molluscan breeding programs described in the literature have either utilized mass (individual) selection, in which selection is performed solely based on the phenotypes of individuals (Fjalestad, 2005), or family-based selection, in which selection is performed on the basis of the phenotypic means of families (between-family selection), on deviations of individuals from their respective family means (within-family selection), or a combination of both (combined selection) (Fjalestad, 2005). A number of commercial and experimental molluscan breeding programs have been described in the literature (Table 2).

**Table 2:** Survey of large-scale breeding programs for cultured molluscs.

**Common name**	**Species**	**Group**	**Location**	**Type of selection**	**Program type**	**Founded**	**References**
Black-footed paua	*Haliotis iris*	Abalone	New Zealand	Family; mass	Industrial	2007	Symonds and Heath, 2008; Camara and Symonds, 2014
Small abalone	*Haliotis diversicolor*	Abalone	China	Family	Unknown	2007	Liu et al., 2015
Mediterranean mussel	*Mytilus galloprovincialis*	Mussel	Australia	Family	Industrial	2008	Nguyen et al., 2012
Greenlip mussel	*Perna canaliculus*	Mussel	New Zealand	Family	Industrial	1999	Camara and Symonds, 2014
Pacific oyster	*Crassostrea gigas*	Oyster	USA	Family	Industrial	1996	Langdon et al., 2003; de Melo et al., 2016
Pacific oyster	*Crassostrea gigas*	Oyster	Australia	Family; mass	Industrial	1997	Ward et al., 2005; Kube et al., 2011
Pacific oyster	*Crassostrea gigas*	Oyster	New Zealand	Family	Industrial	1999	Camara and Symonds, 2014
Pacific oyster	*Crassostrea gigas*	Oyster	France	Mass	Experimental	2009	Dégremont et al., 2015
Pacific oyster	*Crassostrea gigas*	Oyster	China	Mass	Experimental	2007	Li et al., 2011; Wang et al., 2012; Zhong et al., 2016
Sydney rock oyster	*Saccostrea glomerata*	Oyster	Australia	Family; mass	Industrial	1990	Nell et al., 1996, 1999; Dove et al., 2013
Bay scallop	*Argopecten irradians*	Scallop	China	Mass	Unknown	2001	Zheng et al., 2004, 2006

*Breeding programs were identified from a review of the literature, and include both experimental and commercial breeding programs. Program type refers to whether the breeding program is integrated with industry (industrial), for experimental purposes (experimental), or unknown*.

There are two large-scale, commercially-integrated selective breeding programs for mussel species: a family-based breeding program for blue mussel (*Mytilus galloprovincialis*) in Australia and a family-based program for greenlip (GreenShell™) mussel (*Perna canaliculus*) in New Zealand. The breeding program for blue mussel was initiated in Victoria, Australia in 2008 with a founder population of 74 full-sib families (Nguyen et al., 2011). In a trial where individuals were selected based on breeding values for total weight, shell shape, and meat yield, the realized selection responses in these three traits were positive and measured 10% for total weight, 3.0% for shell shape, and 2.3% for meat yield (Nguyen et al., 2014).

Centralized, family-based breeding programs for Pacific oyster have been established in Australia (Ward et al., 2005; Kube et al., 2011), New Zealand (Camara and Symonds, 2014), and the United States (Langdon et al., 2003; de Melo et al., 2016), and experimental mass selection has been reported in France (Dégremont et al., 2015) and China (Li et al., 2011). In a comprehensive analysis, a family-based selection program on the west coast of the United States reported accumulative gains for survival and yield of 15.7 and 19.0%, respectively, following five generations of selection (de Melo et al., 2016).

Recent outbreaks of pathogens such as OsHV-1 in New Zealand (Camara and Symonds, 2014) and France (Dégremont, 2011) have led to increased attention on selective breeding for disease resistance in Pacific oyster. Promisingly, an experimental mass selection program in France demonstrated a 61.8% gain in survival over unselected controls in response to OsHV-1 exposure after four generations (average 15.5% per generation; Dégremont et al., 2015). However, since selection was performed without inbreeding control, genetic gain is likely to be less in a long-term program that minimized inbreeding.

A selection of the main commercial and experimental molluscan breeding programs that have been described in the literature is summarized in Table 2.

## Genomic resources

The application of genomic tools to selective breeding in aquaculture involves two processes: the development of genomic resources for key species and the application of those tools toward selective breeding. In general, the current state of most important species are somewhere in between these two steps: genomic resources have been developed but for the most part are not yet applied to selective breeding. The following section contains a survey of genome scale resources underpinning selective breeding in the main farmed mollusc species. Here, we discuss whole genome sequences, genetic linkage maps, genetic marker panels, and identification of quantitative trait loci (QTL) for key production traits.

## Whole genome sequences

Declining costs of next-generation DNA sequencing has made it possible to sequence the entire genome of nearly any species in a cost-effective manner. Genome sequences allow genetic markers to be mapped to a specific location in the genome, and when genetic markers associated with particular traits are identified, a genome sequence provides the opportunity to investigate nearby genes or genetic elements to potentially identify causative mutations. As of 2017, there were 22 published, scaffold-level genome sequences for finfish (Yue and Wang, 2017) and at the time of writing only six for molluscs: Pacific oyster (Zhang et al., 2012), pearl oyster (Takeuchi et al., 2012, 2016), Yesso scallop (Wang et al., 2017), Zhikong scallop (Li et al., 2017), Manila clam (Mun et al., 2017), and Eastern oyster (NCBI accession GCA_002022765.4) with a seventh species, blue mussel, (Nguyen et al., 2014; Murgarella et al., 2016) currently at contig level (Supplemental File 2). While at the time of writing it is currently unpublished, the most complete molluscan genome assembled to date is that of the Eastern oyster (NCBI accession GCA_002022765.4). The assembly was performed using 87x long-read PacBio sequencing technology, and the genome was able to be assembled to chromosome level (https://www.ncbi.nlm.nih.gov/assembly/GCF_002022765.2/).

To avoid problems with genome assembly caused by high levels of polymorphism, mollusc genomes have typically been assembled using highly inbred individuals with reduced heterozygosity (Zhang et al., 2012; Wang et al., 2017) or have used modified assembly techniques to account for excess heterozygosity (Takeuchi et al., 2016; Li et al., 2017). As expected, resequencing whole genomes of wild individuals has revealed extremely high diversity at the nucleotide level. The overall rate of SNP and indel polymorphism in a single wild individual was 1.3% for Pacific oyster, 0.81% for Zhikong scallop, and 1.04% for Yesso scallop, which is six to ten times higher than that observed in humans (Zhang et al., 2012; Li et al., 2017; Wang et al., 2017).

One important insight gained from sequencing molluscan genomes is the observation of lineage-specific expansions in gene families related to immune and stress response (Takeuchi et al., 2012, 2016; Zhang et al., 2012; Guo et al., 2015; Li et al., 2017; Mun et al., 2017; Wang et al., 2017), which may be a key evolutionary innovation related to surviving as sessile organisms in often heterogeneous conditions. An illustrative example is the case of heat shock proteins (HSP), which among other functions help to prevent misfolding of proteins under various types of stress (Parsell and Lindquist, 1993). The Pacific oyster genome contains a remarkable 88 heat shock protein 70 (Hsp70) genes, of which 71 clustered together in a phylogenetic analysis, consistent with a species/taxa specific gene expansion (Zhang et al., 2012). HSPs have been found to be upregulated in multiple mollusc species in response to a variety of stressors, including heat-shock (Li et al., 2012; Zhang et al., 2012), viral challenge (He et al., 2015), and exposure to pollutants (Gao et al., 2007), and the expansion of HSP gene families in molluscan lineages suggests that copy number variation in stress-related genes may be an important factor to exploit for selective breeding. Other gene families showing expansion include the C1q gene which has a role in pathogen detection and the activation of the complement system in the innate immune response and has a 12-fold higher copy number in the Manila clam than in humans (Mun et al., 2017). In Zhikong scallop, 270 gene families were found to be significantly expanded relative to other bivalves, including genes involved in neurotransmission, immune responses, signal transduction and xenobiotic metabolism (Li et al., 2017). As copy number variation is increasingly associated with variation in agriculturally important traits (e.g., Wang et al., 2015), further consideration of gene expansions in molluscs may prove to be a fruitful area of research for selective breeding.

## Genetic linkage maps

Linkage maps provide a framework of the order and spacing of genetic markers and serve as a starting point for the mapping of quantitative trait loci (QTL) for traits of commercial interest and for the development of marker assisted selection (MAS) programs (Liu and Cordes, 2004). First generation maps typically have been based on amplified fragment length polymorphism (AFLP) markers and/or microsatellites, and whereas dense linkage maps for major species such as Pacific oyster typically have been developed through multiple versions or iterations (Hedgecock et al., 2015 and references therein), equally dense, first generation linkage maps for less developed species have been generated de novo using SNP markers (Jones et al., 2013). Table 3 provides information on high density linkage maps developed recently for major farmed species.

**Table 3:** Select linkage maps for mollusc species.

**Common name**	**Species**	**Sex**	**Marker type**	**Markers**	**Marker interval (cM)**	**LGs**	**References**
Triangle pearl mussel	*Hyriopsis cumingii*	Combined	SNP (Msat)	4508 (475)	1.81	19	Bai et al., 2015, 2016
Pearl oyster	*Pinctada fucata*	Combined	SNP	1373	1.41	14	Li and He, 2014
Pacific oyster	*Crassostrea gigas*	Combined	SNP (Msat)	607 (49)	1.4	10	Hedgecock et al., 2015
Silver-lipped pearl oyster	*Pinctada maxima*	Combined	SNP	887	0.94	14	Jones et al., 2013
Pacific oyster x Portuguese oyster	*Crassostrea gigas x Crassostrea angulata*	Combined	SNP	1695	0.8	10	Wang et al., 2016
Zhikong scallop	*Chlamys farrei*	Male/female	SNP	1861/2025	0.62/0.59	19	Jiao et al., 2014
Small abalone	*Haliotis diversicolor*	Combined	SNP	3717	0.59	16	Ren et al., 2016
Akoya pearl oyster	*Pinctada martensii*	Combined	SNP	3117	0.39	14	Shi et al., 2014
Manila clam	*Ruditapes philippinarum*	Combined	SNP	9658	0.42	18	Nie et al., 2017

*A more complete survey is presented in Supplementary File 2*.

### Genetic marker panels for parentage assignment

The control of inbreeding in hatchery-based selective breeding programs is critical and requires collection of reliable pedigree information for brood stock. Physical tagging is one method that allows pedigree information to be retained, but it requires families to be reared separately until they are sufficiently large to tag. This requires substantial and expensive infrastructure which may constrain mating designs to a sub-optimal number of families (Vandeputte et al., 2004). Genetic markers can overcome these limitations and allow pedigrees to be constructed in groups of mixed families regardless of family structure.

Microsatellites have been used for parentage assignment since the 1990s. Eight to Fifteen microsatellite loci typically provide >99 percent assignment power for finfish (Vandeputte and Haffray, 2014). The high prevalence of null alleles in molluscs (Hedgecock et al., 2004) adds considerably to the cost of developing and validating parentage assignment panels. For example, after screening 135 microsatellite loci in Australian Blue mussel (*Mytilus galloprovincialis*) just 10 were found to provide consistent PCR amplification across 74 full-sibling families. Only 62.5% of 2,536 offspring tested could be assigned to single parent pairs using the remaining 10 microsatellites (Nguyen et al., 2011), which is well below the accuracy needed to operate a family based breeding program. Multiplex sets of microsatellites are more efficient in terms of genotyping costs and reduce manual error (Guichoux et al., 2011). By excluding null alleles and using 12 microsatellites each with four to 20 alleles in three multiplex assays, assignment success in the King scallop (*Pecten maximus*) reached 97% accuracy (Morvezen et al., 2013).

The adoption of NGS methods for SNP discovery and technological advances in SNP genotyping have resulted in SNPs supplanting microsatellites as the genetic markers of choice for parentage assignment (Anderson and Garza, 2006; Strucken et al., 2016). The high degree of polymorphism in mollusc genomes provides a massive number of SNPs for marker design, although the frequency of null alleles incurs additional costs for panel development as with microsatellites. Protein-coding regions of the Pacific oyster genome contain one SNP for every 60 bp sequenced compared to one every 40 bp in non-coding regions (Sauvage et al., 2007). SNP density in the European flat oyster was shown to be similarly high with one SNP in every 76 and 47 bp for coding and non-coding regions, respectively (Harrang et al., 2013). Nguyen et al. (2014) reported that in *Mytilus galloprovincialis* 179 SNPs could correctly assign 92.5% of offspring to 77 full-sibling families. Several other SNP-based genotyping panels have recently been published for key species (Table 4).

**Table 4:** Select marker panels for mollusc species.

**Common name**	**Species**	**Markers**	**Marker type**	**Assay type**	**References**
South African abalone	*Haliotis midae*	234	SNP	Illumina GoldenGate	Bester-Van Der Merwe et al., 2013
Silver-lipped pearl oyster	*Pinctada maxima*	2782	SNP	Illumina Infinium	Jones et al., 2013
Pacific oyster	*Crassostrea gigas*	384	SNP	Illumina Goldengate	Lapégue et al., 2014
European flat oyster	*Ostrea edulis*	384	SNP	Illumina Goldengate	Lapégue et al., 2014
Blue mussel	*Mytilus galloprovincialis*	227	SNP	Sequenom iPLEX	Nguyen et al., 2014
Pacific oyster	*Crassostrea gigas*	40,625	SNP	Affymetrix Axiom	Gutierrez et al., 2017
European flat oyster	*Ostrea edulis*	14,950	SNP	Affymetrix Axiom	Gutierrez et al., 2017
Pacific oyster	*Crassostrea gigas*	190,420	SNP	Affymetrix Axiom	Qi et al., 2017

*A more complete survey is presented in Supplementary File 2*.

### Quantitative trait loci

Most economically important traits in aquaculture production are influenced by a large number of small-effect loci, known as quantitative trait loci (QTL). QTL mapping is the process of identifying genetic marker alleles that segregate non-randomly with QTL and positioning those loci in the genome (Lynch and Walsh, 1998). Once QTL for a trait are identified, individuals can be selected for breeding on the basis of marker alleles that segregate with favorable phenotypes (Lande and Thompson, 1990). This strategy, known as marker-assisted selection (MAS), is particularly useful for traits that cannot be measured on selection candidates directly, notably disease resistance or meat-quality traits (Sonesson, 2007a).

QTL mapping in molluscs (as well as terrestrial livestock and finfish) has traditionally relied on linkage-based methods of identifying QTL, in which one or more families are genotyped and scored for a trait of interest, and the co-segregation of markers and trait values are observed for each family (Lynch and Walsh, 1998). While this approach has been used successfully to map QTL in cultured molluscs, it has the disadvantages that marker/QTL associations are only necessarily valid in the families that are genotyped and that QTL are typically not able to be mapped with much precision (Hirschhorn and Daly, 2005). The ability to genotype many thousands of SNP loci has now made possible a different statistical approach to identifying loci associated with traits of interest. Genome-wide association studies (GWAS) identify marker-trait associations at the population rather than the family level by identifying genetic markers that are in linkage disequilibrium with QTL (Hirschhorn and Daly, 2005). While GWAS typically requires many more loci than linkage-based QTL mapping, it is able to provide much more precise locations of QTL, and due to massive improvements in genotyping has begun to be applied in aquaculture (Correa et al., 2015; Gutierrez et al., 2015; Tsai et al., 2015; Vallejo et al., 2017).

The majority of QTL mapping studies in mollusc species have focused on growth, although QTL related to disease resistance (Yu and Guo, 2006; Lallias et al., 2009; Sauvage et al., 2010; Harrang et al., 2015), shell pigmentation (Qin et al., 2007; Petersen et al., 2012; Ge et al., 2014; Zhong et al., 2014), sex (Li et al., 2005; Guo et al., 2012; Jiao et al., 2014), and pearl quality (Jones et al., 2014a,b; Bai et al., 2015, 2016) have also been discovered.

## Opportunities for genomics-based selection

Incorporating genomic information into selective breeding programs (hereafter referred to as genomics-based selection) has become an important aspect of genetic improvement in livestock and plant breeding (Meuwissen et al., 2013; Muranty et al., 2014). The principal advantage of incorporating marker information into breeding programs is an increase in the accuracy of predicted breeding values, which in turn causes a proportional increase in the response to selection (Martinez, 2007). Because selection is based on marker genotypes, MAS is useful for traits that are difficult to measure on breeding candidates (Sonesson, 2007b), particularly when markers are linked to large-effect QTL (Robledo et al., 2017). A classic example of MAS in aquaculture is the case of IPN virus resistance in Atlantic salmon, in which a large-effect QTL was discovered in Scottish and Norwegian populations (Houston et al., 2008; Moen et al., 2009), and MAS using markers linked to the locus was subsequently incorporated into commercial breeding programs, substantially reducing the incidence of IPN in commercial facilities (Moen et al., 2015; Robledo et al., 2016).

A more recent development, genomic selection (GS), is the extension of MAS to a genome-wide scale. Rather than targeting specific QTL, GS assumes that genetic markers (typically SNPs) are genotyped at sufficient density such that all QTL are in linkage disequilibrium with at least one marker (Meuwissen et al., 2001), and so in theory can utilize all of the genetic variance in a complex trait (Meuwissen et al., 2013). In practice, a reference population of individuals with both genotypes and phenotypes is used to estimate the effects of each marker on the phenotype of interest, and these effects are used to estimate breeding values for relatives of the reference population (selection candidates) that have been genotyped but have no phenotypes (Meuwissen et al., 2001). Whereas MAS is useful when large-effect loci are present, GS is well-suited for improvement of traits that are under the control of many loci with small effect (Robledo et al., 2017).

MAS and GS have yet to be applied in breeding programs for molluscs, although several studies in other aquaculture species have demonstrated increases in accuracy of breeding value predictions for traits such as growth (Tsai et al., 2015; Nguyen et al., 2018) and disease resistance (Bangera et al., 2017; Robledo et al., 2018). In molluscs there has been significant investment in identifying QTL, and large-effect loci may be applicable to MAS. For example, a QTL explaining as much as 80% of the phenotypic variance for shell pigmentation has been identified in Pacific oyster (Ge et al., 2014). Reduced-representation sequencing techniques such as GBS or RAD-seq will likely be important for future genomics-based selection in molluscs, as such techniques allow for pedigree reconstruction, estimation of trait heritability, genetic map construction, identification of QTL, and estimation of genomic breeding values in the same experiment (Palaiokostas et al., 2016; Robledo et al., 2017). QTL mapping studies involving GBS or RAD-seq have been published for scallops (Jiao et al., 2014), oysters (Li and He, 2014; Shi et al., 2014; Wang et al., 2016), clams (Nie et al., 2017), and abalone (Ren et al., 2016). Recently, the feasibility of GS using 2b-RAD (Wang et al., 2012) was demonstrated in an experiment involving Yesso scallops (*Patinopectin yessoensis*; Dou et al., 2016). However, while such techniques have considerably reduced the time and expense involved in generating the genomic resources necessary for MAS or GS, the cost of genotyping is still a significant barrier toward further use of these strategies.

Challenges for incorporating genomic approaches in breeding of molluscs include high rates of larval mortality, high incidence of marker segregation distortion, and self-fertilization. A better theoretical understanding of these processes in molluscs will lead to the development of strategies for more optimally balancing control of inbreeding and genetic gain. In addition, the transition from low to high-throughput genotyping technologies will alleviate marker-specific issues such as local segregation distortion, because these markers can be removed from a large dataset with minimal impact on the results. The lack of high quality annotated reference genomes and studies to understand the genetic architecture of important production traits for key species is also a bottleneck for the adoption of genomics-based breeding. Only three of the top ten cultivated molluscan species (Table 1) have had their genomes sequenced to draft level, and the high degree of variability is still a barrier to developing accurate assemblies. Although a reference genome sequence is available for Pacific oyster (Zhang et al., 2012), a significant number of putative assembly errors have been identified (Hedgecock et al., 2015). The low economic value of individual animals is also a challenge for implementation of genomic selection, and novel methods that can reduce the cost of genotyping or reduce the number of markers necessary for genomic selection will be necessary to make this a cost-effective strategy. The development of medium- and high-density SNP arrays for important species, such as those recently developed for Pacific oyster and European flat oyster (Gutierrez et al., 2017) and Pacific oyster (Qi et al., 2017), will make genomic selection more feasible by enabling high-throughput genotyping at low cost per marker. In addition, several ideas have been proposed to reduce genotyping costs for MAS and GS in aquaculture applications, including pooling DNA of test individuals (Sonesson et al., 2010), using low-density genotyping for within-family genomic selection (Lillehammer et al., 2013), and imputation of genotypes using a combination of low- and high-density genotyping (Tsai et al., 2017).

Providing these bottlenecks can be overcome, the gains in accuracy of breeding value predictions that can be obtained using genomic data, along with optimized control of inbreeding, should enable more rapid rates of genetic improvement in cultured molluscs compared to traditional selection schemes.

## Author contributions

All authors listed have made a substantial, direct and intellectual contribution to the work, and approved it for publication.

### Conflict of interest statement

IJ is Chief Executive Officer of Xelect Ltd. and CH is a consultant for Xelect Ltd.
